# Analysis of genome sequence and symbiotic ability of rhizobial strains isolated from seeds of common bean (*Phaseolus vulgaris*)

**DOI:** 10.1186/s12864-018-5023-0

**Published:** 2018-08-30

**Authors:** Alejandro Aguilar, Yolanda Mora, Araceli Dávalos, Lourdes Girard, Jaime Mora, Humberto Peralta

**Affiliations:** 1 Functional Genomics of Prokaryotes, Center for Genomic Sciences, National University of Mexico, Av. Universidad, CP 62210 Cuernavaca, Morelos Mexico; 2 Genome Dynamics, Center for Genomic Sciences, National University of Mexico, Av. Universidad, CP 62210 Cuernavaca, Morelos Mexico; 3 Systems Biology and Synthetic Biology, Center for Genomic Sciences, National University of Mexico, Av. Universidad, CP 62210 Cuernavaca, Morelos Mexico

**Keywords:** Non-symbiotic rhizobia, Endophyte, Evolution, Symbiosis

## Abstract

**Background:**

Rhizobia are alpha-proteobacteria commonly found in soil and root nodules of legumes. It was recently reported that nitrogen-fixing rhizobia also inhabit legume seeds. In this study, we examined whole-genome sequences of seven strains of rhizobia isolated from seeds of common bean (*Phaseolus vulgaris*).

**Results:**

Rhizobial strains included in this study belonged to three different species, including *Rhizobium phaseoli*, *R. leguminosarum*, and *R. grahamii.* Genome sequence analyses revealed that six of the strains formed three pairs of highly related strains. Both strains comprising a pair shared all but one plasmid. In two out of three pairs, one of the member strains was effective in nodulation and nitrogen fixation, whereas the other was ineffective. The genome of the ineffective strain in each pair lacked several genes responsible for symbiosis, including *nod*, *nif*, and *fix* genes, whereas that of the effective strain harbored the corresponding genes in clusters, suggesting that recombination events provoked gene loss in ineffective strains. Comparisons of genomic sequences between seed strains and nodule strains of the same species showed high conservation of chromosomal sequences and lower conservation of plasmid sequences. Approximately 70% of all genes were shared among the strains of each species. However, paralogs were more abundant in seed strains than in nodule strains. Functional analysis showed that seed strains were particularly enriched in genes involved in the transport and metabolism of amino acids and carbohydrates, biosynthesis of cofactors and in transposons and prophages. Genomes of seed strains harbored several intact prophages, one of which was inserted at exactly the same genomic position in three strains of *R. phaseoli* and *R. leguminosarum*. The *R. grahamii* strain carried a prophage similar to a gene transfer agent (GTA); this represents the first GTA reported for this genus.

**Conclusions:**

Seeds represent a niche for bacteria; their access by rhizobia possibly triggered the infection of phages, recombination, loss or gain of plasmids, and loss of symbiosis genes. This process probably represents ongoing evolution that will eventually convert these strains into obligate endophytes.

**Electronic supplementary material:**

The online version of this article (10.1186/s12864-018-5023-0) contains supplementary material, which is available to authorized users.

## Background

Rhizobia belong to the group of alpha-proteobacteria and thrive in diverse environments, either as free-living bacteria in soil or as symbionts within the root nodules of legumes, where they fix atmospheric nitrogen [1]. Rhizobia have also been isolated from other tissues of legumes, such as leaves, roots, and stems [2], and from other plants and trees, including *Triticum aestivum* [3], *Zea mays* [4], *Oryza sativa* [5], *Arabidopsis thaliana* [6], and *Populus euphratica* [7].

Recently, we showed that nitrogen-fixing rhizobia also occur within the seeds of common bean (*Phaseolus vulgaris*) [8]; this expands the range of sites populated by rhizobia. Moreover, the presence of rhizobia within the seeds possibly represents a new beneficial interaction between the host plant and the microsymbiont, as these bacteria can be propagated simply with seed germination. We have shown that these bean seeds contain various strains of *R. phaseoli*, *R. leguminosarum*, *R. grahamii*, and *Sinorhizobium americanum*. Some of these strains form pairs with other strains based on the plasmid profiles; however, only one of the members comprising the pair is capable of fixing nitrogen [8]. We performed genomic analysis of some of these strains to search for genes and their functions that enable these bacteria to inhabit the bean seeds [9].

High similarity has also been shown among strains of *Agrobacterium pusense* isolated from root nodules of un-inoculated bean plants; the source of these bacteria is thought to be seeds [10]. These *Agrobacterium* strains are closely related to the strain IRBG74, which is currently the only *Agrobacterium* isolate with a symbiotic plasmid, capable of forming nitrogen-fixing root nodules in *Sesbania cannabina* [11, 12]. Notably, some of these bacterial strains contain several prophages, which can serve as markers of the arrival of these strains in their new environment. Phages help bacteria expand their niche through the transfer and acquisition of genes [13].

It is important to determine the genomic sequences of these rhizobial strains and to identify the sequence variation resulting from the loss of symbiotic ability in some of them, as this information would help elucidate the process of evolution and functional consequences of changes in gene content. In this study, we report the genomic sequences of seven rhizobial strains isolated from the interior of bean seeds. We analyzed these sequences to understand the modifications in chromosomes, plasmids, orthologous gene sequences, and gene functions that possibly emerged as a consequence of the arrival of these strains in the seeds.

## Results

We analyzed the genomes of seven rhizobial strains isolated from nodules of un-inoculated common bean plants. Characteristics of these strains and of those used for comparisons are summarized in Table 1. The genomes were grouped according to their phylogenetic relatedness reported previously [8]. These seven strains were assigned to three species: *R. phaseoli* (containing strains CCGM2, CCGM8, and CCGM9), *R. grahamii* (CCGM3), and related to *R. leguminosarum* (CCGM4, CCGM5, and CCGM6). The *R. phaseoli* strain CCGM1 was isolated in the same experiments with the rest of strains and was included in this study for comparison with CCGM2. Both strains form a highly related pair [8].

**Table 1:** Genome features of rhizobial strains used in this study

Strain	Genome size (Mb)	No. of contigs	N50 contigs (bp)	No. of scaffolds	No. of genes	No. of RNAs	%G + C content	GenBank accession no.
*Rhizobium phaseoli*								
CCGM1^a^	6.87	139	129,009	55	6428	61	61.1	JFGP00000000
CCGM2	7.03	53	353,323	19	6834	57	61.3	MZZP00000000
CCGM8	6.47	52	204,711	12	6247	58	61.5	MZZZ00000000
CCGM9	6.92	116	137,996	13	6736	52	61.2	NAAA00000000
CIAT652	6.44	–	–	4^b^	6109	61	61.3	CP001074–77
Related to *Rhizobium leguminosarum*								
CCGM4	7.12	42	355,929	8	6798	59	61.5	NAON00000000
CCGM5	6.91	47	318,800	12	6617	60	61.4	NAOO00000000
CCGM6	7.40	69	356,355	36	7139	58	61.2	NAOP00000000
WSM2304	6.88	–	–	5^2^	6415	60	61.2	CP001191–95
*Rhizobium grahamii*								
CCGM3	7.08	66	288,576	30	6839	53	59.5	NAAC00000000
CCGE502	7.15	80	343,668	80	6844	54	59.4	AEYE00000000

^a^Published previously in [8]. ^b^Complete genomes: chromosome and plasmids

Of the strains analyzed here, four were detected by PCR in seeds harvested from plants inoculated with these strains; these included CCGM1, CCGM2, CCGM3, and CCGM6 (Additional file 1). Targeted genes were strain-specific ones in the case of strains CCGM1 and CCGM2 (a gene for a glycosyl transferase and a gene with hypothetical function, respectively) and *rpoB* (the RNA polymerase beta subunit, an unique copy gene) in the case of CCGM3 and CCGM6. The sequences retrieved from PCR products were 100% identical to the corresponding segment in the sequenced strains. The strains CCGM4 and CCGM8 did not present symbiotic plasmids; and thus, plants inoculated with them will not yield seeds. Strain CCGM5 was not detected in seeds. Strain CCGM9 was not included in the greenhouse assay.

### Comparison of plasmid profiles and gene content

**Fig. 1:**
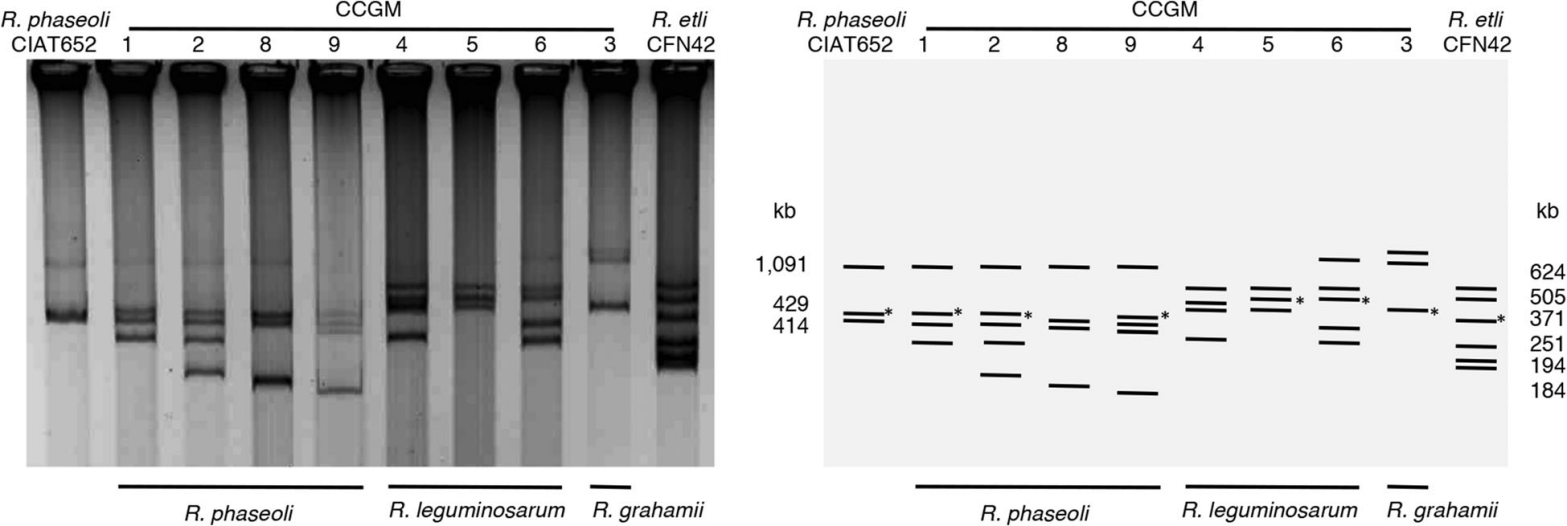
Plasmid profiles of rhizobial strains. Left, the image shows *R. phaseoli* strains CCGM1, CCGM2, CCGM8, and CCGM9, strains CCGM4, CCGM5, and CCGM6 related to *R. leguminosarum*, and *R. grahamii* strain CCGM3. *R. phaseoli* strain CIAT652 and *R. etli* strain CFN42, with plasmids of known size, are also shown. Plasmids were visualized using the Eckhardt technique. Right, graphic summary of detected plasmids. Asterisks denote symbiotic plasmids detected by Southern hybridization in [8], with exception of CCGM9, which was assigned comparing genome assembly with CCGM8

Some seed strains presented almost identical plasmid profiles, as shown in Fig. 1 and as reported previously [8]. Notably, shared plasmids generally contained the same genes and genome sequences of highly related strains were approximately 99% identical in the sequence of shared genes. Therefore, these pairs of strains may be considered almost as clones that have diverged very recently, mainly because of insertions or deletions of genes.

The *R. phaseoli* group had two pairs of highly related strains. The first pair comprised the strains CCGM8 and CCGM9, which shared three plasmids; however, a fourth plasmid (~ 400 kb) was present only in CCGM9 (Fig. 1). This fourth plasmid was the symbiotic plasmid, because it contained several genes involved in symbiosis. The CCGM9 strain harbored 497 additional genes compared with CCGM8 (Additional file 2); this difference in the number of genes was in agreement with the size of the distinguishing plasmid. The second pair comprised the strains CCGM1 and CCGM2. Both these strains shared four plasmids. However, a fifth plasmid (~ 160 kb) was present only in CCGM2 (Fig. 1). The CCGM2 strain harbored an additional 406 genes compared with CCGM1 (Table 1). The number of these additional genes was excessive for the size of the fifth unshared plasmid, suggesting that more gene insertions occurred either in the other four plasmids or in the bacterial chromosome. The list of genes differing in both strains is shown in Additional file 2. Both strains presented complete sets for nodulation and nitrogen fixation.

In strains related to *R. leguminosarum*, one highly related pair was observed (strains CCGM4 and CCGM5). Both these strains shared the same plasmid profile, with the exception of a 240 kb plasmid, which was present only in CCGM4 (Fig. 1). The genome of CCGM4 carried 297 more genes than that of CCGM5 (Additional file 2), which was in agreement with the size of the additional plasmid. On the other hand, CCGM5 had 118 genes not present in CCGM4 mainly in two contigs, which carried several symbiotic genes (Additional file 2).

### Structural analysis of genomes

#### *R. phaseoli* strains

**Fig. 2:**
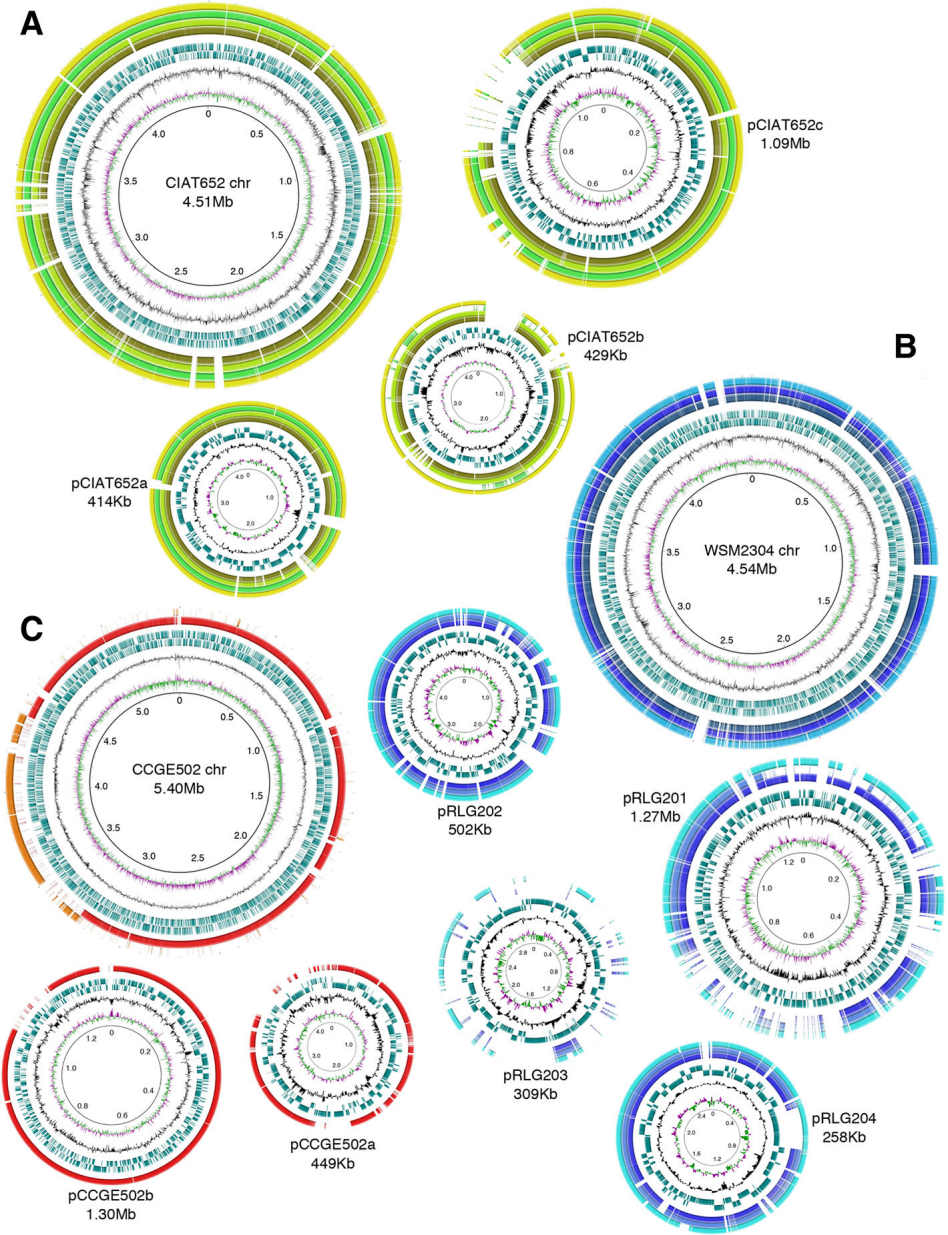
Structural comparisons among genomes (chromosome and plasmids) of seed and nodule rhizobial strains. **a** *Rhizobium phaseoli*. The genome of the nodule strain CIAT652 was used as a reference for comparisons with seed strains of this species. Predicted open reading frames (ORFs) of seed strains with homology to CIAT652 are indicated: CCGM1 (dark green), CCGM2 (lime), CCGM8 (green), and CCGM9 (yellow). **b** Strains related to *R. leguminosarum*. The genome of the nodule strain WSM2304 was used as a reference for comparisons with seed strains of this species. Predicted ORFs of seed strains with homology to WSM2304 are indicated: CCGM4 (dark blue), CCGM5 (lead blue), and CCGM6 (sky blue). **c** *R. grahamii*. The genome of the nodule strain CCGE502 was used as a reference for comparison with the seed strain CCGM3. Predicted ORFs of CCGM3 with homology to CCGE502 are shown in red. The homology between a CCGM3 contig (possibly a plasmid) and the CCGE502 chromosome is indicated in orange. For each replicon, different features are indicated starting from the innermost circle, including G + C skew for predicted ORFs, G + C content of ORFs (%; black), and predicted ORFs in the plus or minus strands (turquoise)

The comparison of genomes of seed rhizobia with that of the nodule strain CIAT652 revealed high level of conservation between chromosomes (approximately 98% in nucleotide identity) (Fig. 2). However, some small chromosomal fragments were absent from the seed strains. As observed, some of these fragments exhibited low GC content. The nodule strain CIAT652 contains three plasmids, namely, pCIAT652a, pCIAT652b (pSym), and pCIAT652c. The sequence of pCIAT652a was almost completely conserved among the seed strains, with the exception of three small fragments. In the case of pCIAT652b, the symbiotic plasmid, most of the seed strains appeared to lack two small fragments; however, the CCGM8 strain lacked this plasmid almost completely. Majority of the plasmid pCIAT652c (~ 1 Mb) was well conserved in the seed strains, with the exception of a 200 kb fragment. Strains CCGM8 and CCGM9 lacked additional small fragments as well.

#### Strains related to *R. leguminosarum*

Seed strains of this group were compared with the nodule strain WSM2304, which contains four plasmids, pRLG201, pRLG202, pRLG203, and pRLG204. The seed strains CCGM4, CCGM5, and CCGM6 showed high sequence similarity with the chromosome sequence of WSM2304 (92% in nucleotide identity), with the exception of some segments that were absent from all seed strains (Fig. 2). Plasmid pRLG201, the symbiotic plasmid, is the largest among the four plasmids; only half of this plasmid was conserved in the seed strains. The first quarter absent in CCGM5 represents the lacking plasmid compared with CCGM4. Plasmids pRLG202 and pRLG204 were well conserved among the seed strains; however, one segment of the pRLG204 was absent from the strains CCGM4 and CCGM5. Sequences of the plasmid pRLG203 were scarcely shared with the seed strains.

#### *R. grahamii* strains

The strain for comparison was the only available genome of the species, that of CCGE502. It contains two plasmids, pCCGE502a (the symbiotic plasmid, 449 kb) and pCCGE502b (1.3 Mb). However, the seed strain CCGM3 contained three plasmids, two of which were similar to pCCGE502a and pCCGE502b, whereas the third plasmid appeared to share homology with a 1 Mb chromosomal segment of CCGE502 (Fig. 2).

### Orthologs and paralogs in the seed strains

**Fig. 3:**
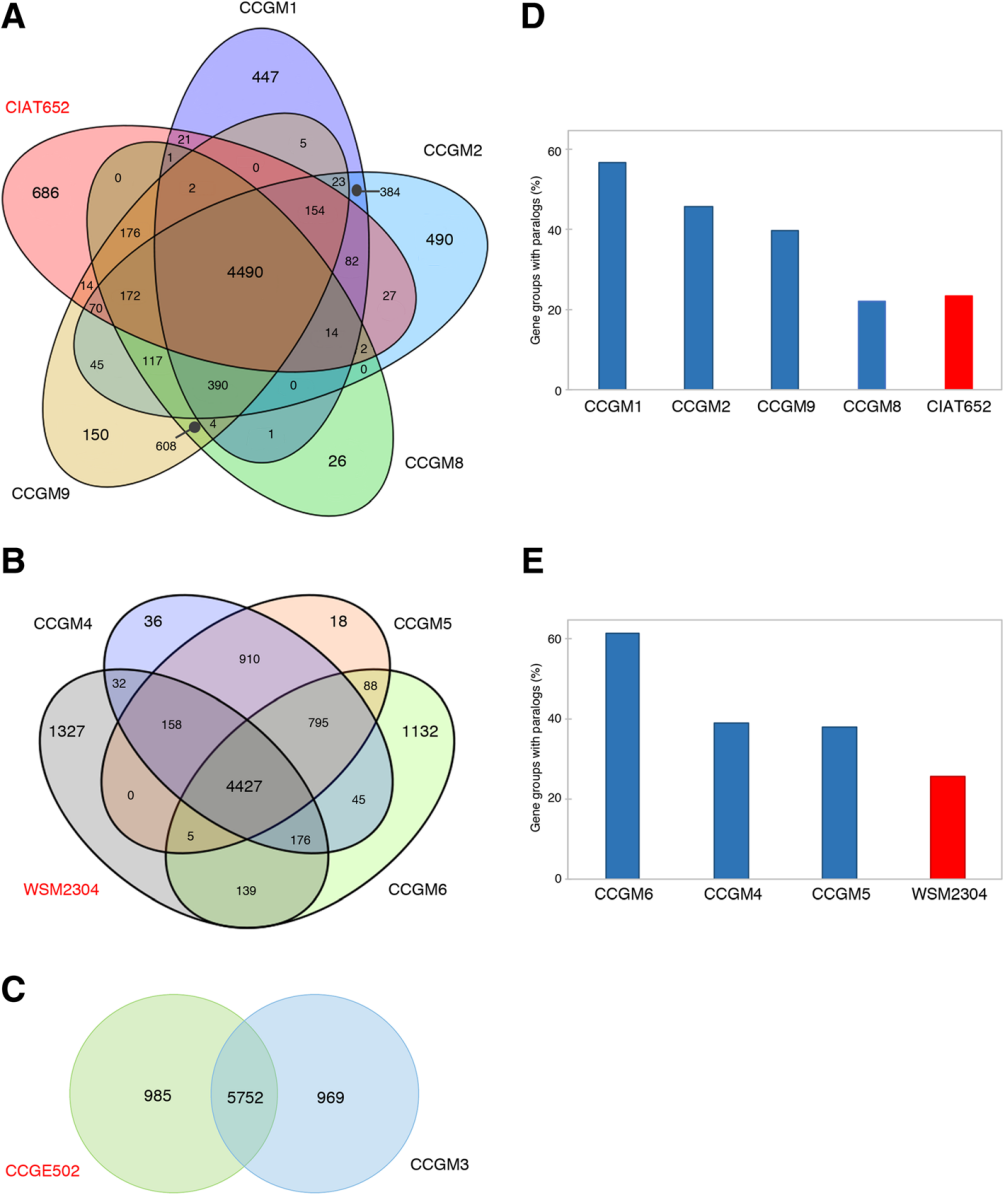
Detection of orthologs and paralogs in genomes of rhizobial strains. Comparisons of the number of orthologs between (**a**) *R. phaseoli* nodule strain CIAT652 and seed strains CCGM1, CCGM2, CCGM8, and CCGM9, (**b**) strain WSM2304, related to *R. leguminosarum,* and seed strains CCGM4, CCGM5, and CCGM6, and (**c**) *R. grahamii* nodule strain CCGE502 and seed strain CCGM3. Abundance of paralogs in (**d**) *R. phaseoli* strains and (**e**) related to *R. leguminosarum*

A number of genes were similar among the rhizobial strains. In the *R. phaseoli* group, 4490 genes (approximately 70% of the total) were orthologous among the strains (Fig. 3a). By contrast, only 26–686 genes were strain-specific. Because of the high sequence similarity between the strains CCGM8 and CCGM9, the number of specific genes was very reduced; the pair shared 608 genes, presented exclusively in the two strains. The strains related to *R. leguminosarum* shared 4427 orthologous genes, and the number of strain-specific genes ranged from 18 to 1327. The pair CCGM4 and CCGM5 shared 910 genes, presented exclusively in the two strains (Fig. 3b). In the case *of R. grahamii*, the nodule strain CCGE502 and the seed strain CCGM3 shared 5752 genes, and 985 and 969 genes were specific to CCGE502 and CCGM3, respectively (Fig. 3c).

We previously reported an increase in the number of paralogous sequences in seed strains [9]. In this study, we found 257 and 187 clusters of genes with paralogs in *R. phaseoli* and *R. leguminosarum*, respectively; we evaluated the abundance of paralogs by strain in these clusters. As shown in Fig. 3d, *R. phaseoli* seed strains CCGM1, CCGM2, and CCGM9 contained greater number of paralogs than the nodule strain, CIAT652. Similarly, the seed strains related to *R. leguminosarum,* CCGM4, CCGM5, and CCGM6 harbored more number of paralogs than the nodule strain, WSM2304 (Fig. 3e). *R. grahamii* strains were not evaluated.

To assess the overall sequence homology among strains analyzed here, we calculated the average nucleotide identity (ANI) and genome coverage (Additional file 3). Pairs of highly related strains showed high ANI between their genomes: 99.8% between CCGM1 and CCGM2, 99.9% between CCGM8 and CCGM9, and 99.9% between CCGM4 and CCGM5. The genome coverage varied from 68 to 98% in *R. phaseoli* and from 72 to 100% in strains related to *R. leguminosarum*; the corresponding value was 92% in *R. grahamii*. Notably, seed strains related to *R. leguminosarum* (CCGM4, CCGM5, and CCGM6) showed ANI genomic values of 92% in comparison to the nodule strain; this value was below the threshold level (95%) accepted for assigning a strain to a given species.

### Functional analysis of orthologous and strain-specific genes

**Fig. 4:**
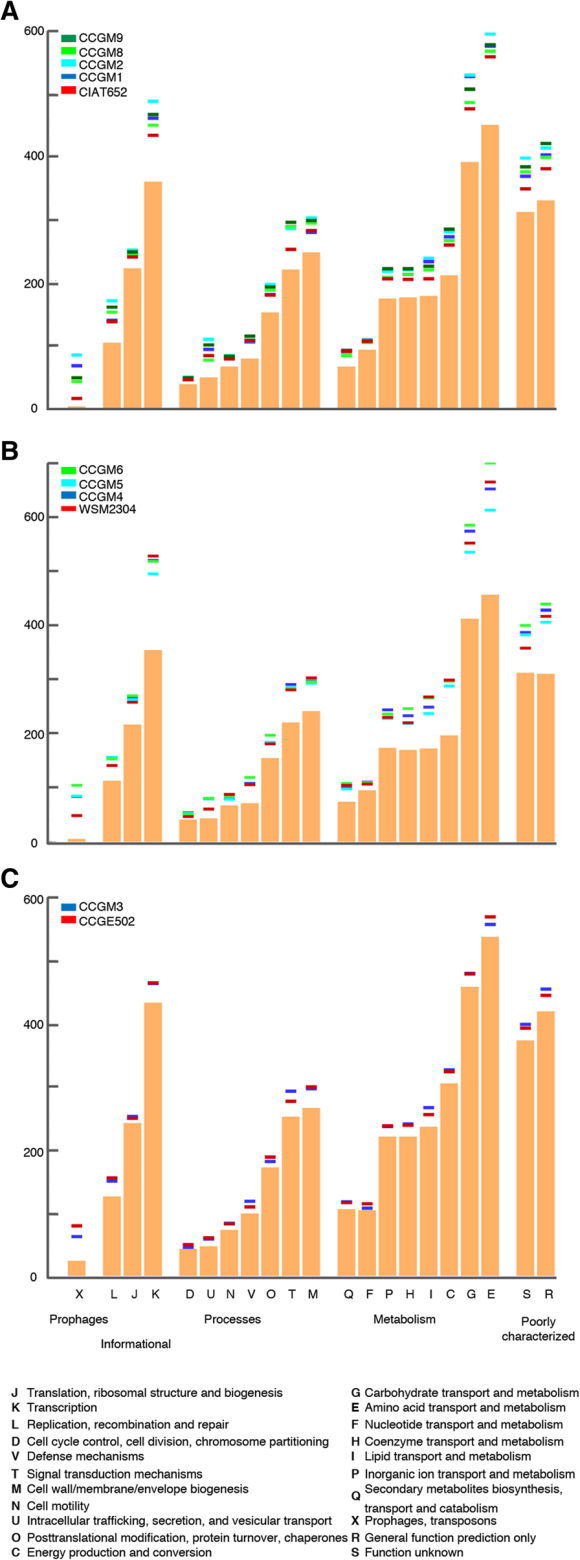
Functional classification of rhizobial genes. Bar graphs show the distribution of functional classes by clusters of orthologous groups (COG) of shared orthologs in (**a**) *R. phaseoli* (4490 genes), (**b**) *R. leguminosarum* (4427 genes), and (**c**) *R. grahamii* (5792 genes). Lines at the top of each graph represent the functional distribution of the genome of each strain

The functional classification of genes, including the shared orthologs, of rhizobial strains of each group is shown in Fig. 4. The high similarity in the functional classification of genes between different groups was noteworthy. In the three species, important number of genes were involved in the transport and metabolism of carbohydrates (G) and amino acids (E). By contrast, class X, comprising prophages and transposases, showed the lowest number of orthologs.

To identify genes that were potentially important for the survival of seed rhizobia, functions of genes present only in seed strains were compared with those present only in nodule strains (Additional files 4 and 5). Some functions were significantly enriched (*p* < 0.05, Fisher’s exact test) in seed strains belonging to different groups. For example, transcription (class K), intracellular trafficking (U), carbohydrate metabolism (G), amino acid metabolism (E), and synthesis of cofactors (H) were enriched in *R. phaseoli*; intracellular trafficking (U) and synthesis of cofactors (H) in strains related to *R. leguminosarum*; and prophages and transposases (X) in *R. grahamii*. By contrast, the nodule rhizobia were enriched in genes of unassigned function (not in COGs) and in *R. grahamii* CCGE502 also in defense (V).

We found several genes in the seed strains possibly related to their living style. In *R. phaseoli* CCGM1 we observed genes such as extensin, porin, rhizobiocin, cellulose synthases and cellulosome anchoring system (Additional file 5). In CCGM2 genes like adhesin, phasin, and several conjugal transfer genes (Additional file 5). The pair CCGM8 and CCGM9 represented a special case because they were isolated from late nodules of un-inoculated plants, irrigated with nitrate and ammonium [8]. Among the strain-specific genes harbored by the pair we found several belonging to nitrite and nitrate assimilation such as nitrate reductase, nitrite reductase, nitric oxide reductase, and their transcriptional regulators (Additional file 5). The pair CCGM4 and CCGM5 had specific genes such as trehalose phosphatase, cupins, pyoverdin, and phasin. In CCGM6 we found adhesins, aquaporin, cupins, luciferase, patatin, and several *vapC*-related toxins (Additional file 5).

In order to assess the functional reiteration, we analyzed name genes with reiterated function in the orthologs and strain-specific genes (Additional file 6). More abundant functional reiteration was found in nodule strains of *R. phaseoli* and the strain related to *R. leguminosarum*; in contrast, in *R. grahamii* the seed strain presented more reiteration.

### Detection of prophages

**Fig. 5:**
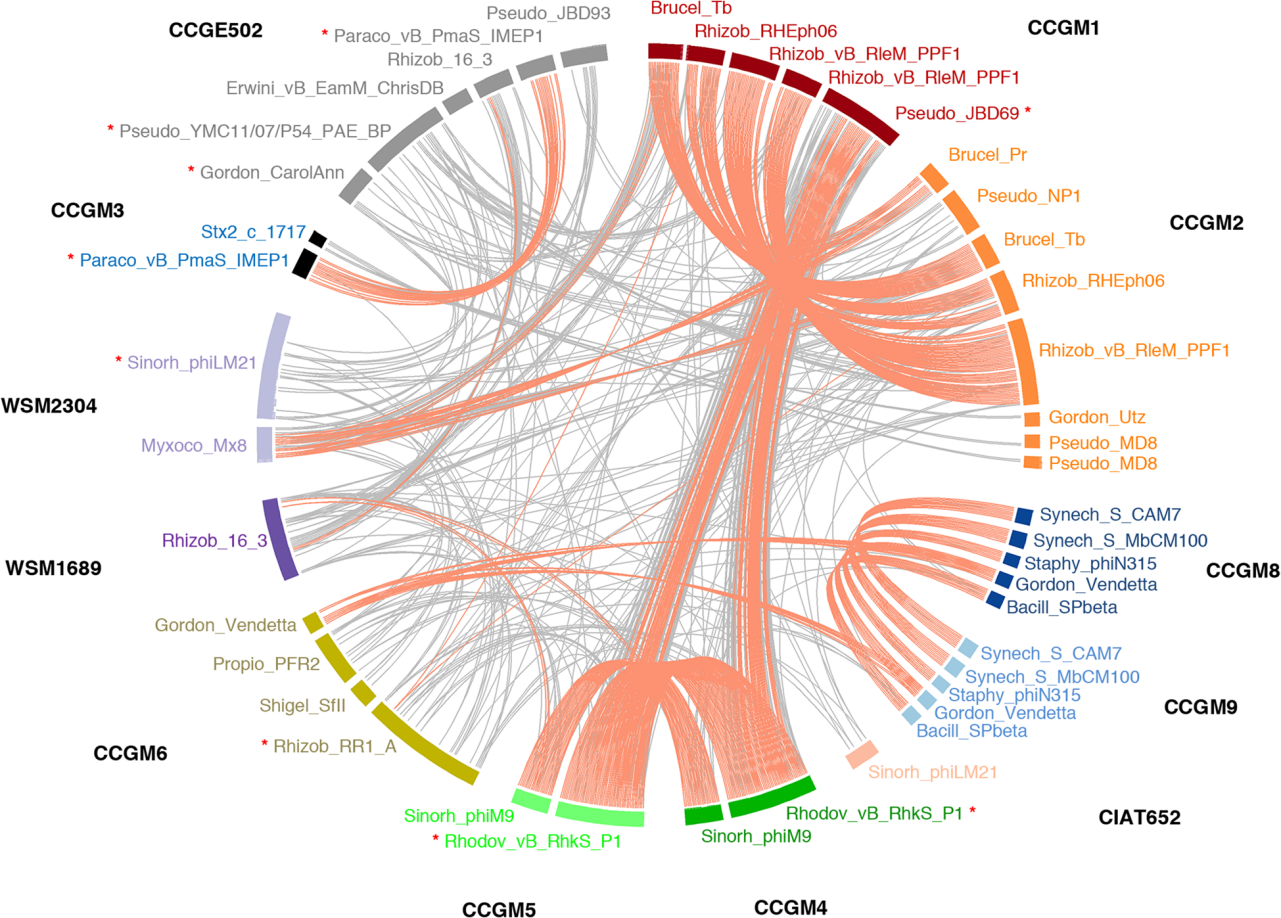
Predicted prophages in rhizobial genomes. Thick lines forming the circle represent predicted prophages. Rhizobial strains harboring these prophages are indicated with different line colors: *R. phaseoli* strains CCGM1, CCGM2, CCGM8, CCGM9, and CIAT652 are indicated in red, orange, blue, light blue, and pink, respectively. Strains related to *R. leguminosarum*, CCGM4, CCGM5, CCGM6, WSM1689, and WSM2304 are indicated in dark green, light green, lime, navy blue, and lead blue, respectively. *R. grahamii* strains CCGM3 and CCGE502 are indicated in black and gray, respectively. Lines connecting various prophages within the circle indicate protein homology levels; red lines indicate protein homology ≥90% and gray lines indicate protein homology ≤90%). Intact prophages are indicated with asterisks. For complete data on each prophage, see Additional file 6

Sequence analysis of seed rhizobia isolated in this study revealed the insertion of several prophages in their genomes. A schematic representation of genomic fragments predicted to encode prophages in seed and nodule strains, and the homology of these sequences to those available in databases are shown in Fig. 5 and Additional file 7. The genome of *R. phaseoli* strain CCGM1 harbored five prophages, of which four were related to prophages previously identified in Rhizobiales, such as Rhizob_vB and Brucel_Tb, and one was homologous to a prophage identified in *Pseudomonas* (Pseudo_JBD). The prophage Pseudo_JBD showed high sequence similarity with one prophage of strains related to *R. leguminosarum*, CCGM4 and CCGM5, suggesting that this prophage was transferred to bean seeds at the same time as the rhizobial seed strains. The strain CCGM2 shared three prophages with the strain CCGM1, all of which are commonly found in rhizobia and are related to Brucel_Pr. Strain CCGM2 also had four small genomic fragments related to prophages found in *Gordonia* and *Pseudomonas.* Genomes of strains CCGM8 and CCGM9 carried five identical prophages, and these were related to some of the prophages found in *Synechocystis*, *Bacillus*, *Staphylococcus*, and *Gordonia* (Fig. 5, Additional file 7).

Genomes of seed strains CCGM4 and CCGM5 harbored two identical prophages. One of them (Rhodov_vB) was 92% identical to CCGM1 prophage Pseudo_JBD. The Rhodov_vB prophage was inserted at the same chromosomal position, i.e., in the tRNA-Leu gene, in the strains CCGM4, CCGM5 and CCGM1. The other prophage was almost identical between CCGM4 and CCGM5, and was similar to Sinorh_phiM9. The strain CCGM6 harbored very different prophages, with only one related to Rhizobiales (Rhizob_RRA1) and the others related to prophages found in *Gordonia*, *Propionibacterium*, and *Shigella* (Fig. 5, Additional file 7).

The genome of *R. grahamii* strain CCGM3 harbored two probable prophages. One of these prophages was predicted to be complete (Paraco_vB_pMaS_IMEP1) and belonged to a new class of prophages, referred to as gene transfer agents (GTAs) [14, 15]. Moreover, this prophage was homologous to the prophage of strain CCGE502 (Fig. 5, Additional file 7).

## Discussion

Genomic sequences of seven new seed rhizobial strains were obtained and analyzed in this study. In addition to the genomes of four strains analyzed previously [8, 10], sequence analysis of these seed strains expands our view of the species diversity and characteristics of rhizobia found in bean seeds. The findings reported here shed light on the evolution of these microorganisms in this new niche.

The bioinformatics approach used here was very helpful in detecting the overall relatedness between seed and nodule strains (Fig. 3). These analyses also revealed peculiarities of genome sequences that may be crucial for the survival and maintenance of these strains in the seeds. In general, chromosome sequences were well conserved among the strains; however, plasmid sequences showed clear signs of change and extensive recombination (Fig. 2). The most striking features of rhizobial genomes were the presence or absence of a plasmid and their contrasting symbiotic phenotypes (Fig. 1).

Of the strains examined in this study, six formed three pairs of highly related strains (Fig. 3). A similar analysis of a highly-related pair of *Agrobacterium* strains (CCGM10 and CCGM11) has been published previously [10]. Strains CCGM10 and CCGM11 differ from each other in one plasmid, which represents an intact phage. Both these strains belong to *A. pusense*; only one strain of this species, IRBG74, is capable of forming root nodules in legumes to fix nitrogen [12]. Strains CCGM10 and CCGM11 have been isolated from root nodules of un-inoculated bean plants and are unable to nodulate or fix nitrogen. Additionally, genomes of these strains do not harbor genes involved in symbiosis, other than those atypically found in *Agrobacterium* strains, such as *fixK* or *fixNOQP*.

Some of the seed strains are ineffective in forming root nodules and fixing nitrogen; this raises the question as to how these strains accessed the nodules. We propose that effective rhizobial strains or their clones originally formed these nodules, and the ineffective strains co-inhabited the nodules. The pair of rhizobial strains comprising CCGM1 and CCGM2 possibly represents a special case. Although both these strains were effective in nodulation and nitrogen fixation and showed almost identical genomes, they differed by one plasmid.

Plants can sanction ineffective rhizobial species or their strains with inferior performance [16]. The symbiotic relationship between plants and rhizobia is delicate and can be broken if one of the participants become damaging to the other. We previously showed that activating ammonium assimilation in the nodule through the function of glutamate dehydrogenase has a deleterious effect on the plant [17]. Similarly, the overexpression of an enzyme involved in arginine biosynthesis adversely affects plant health [18]. By contrast, the elimination of the biosynthetic pathway of poly-beta-hydroxybutyrate [19], or the overexpression of nitrogenase genes [20] improves plant growth and seed yield. It is known that addition of sources of reduced nitrogen inhibits the symbiosis [21]; however, the strains CCGM8 and CCGM9 were isolated precisely from late nodules of plants irrigated with nitrate and ammonium and their genomes showed specific genes that allowed them to assimilate such sources of nitrogen. Apparently our procedure helped to select rhizobial populations with particular capabilities.

The determination of ANI was essential for estimating the sequence similarity between strains and for identifying highly related strains (Additional file 3). Values of ANI approximated 100% between strains, indicating that these strains were clones; any differences in their genomes were mainly due to insertions or deletions of genes. Seed strains classified as related to *R. leguminosarum,* based on their 16S rRNA or *rpoB* sequences [8], showed low ANI values (92%). This value is lower than that generally accepted for assigning a strain to a given species (95%) [22, 23]. Using chromosome sequences of rhizobial strains available in the databases, we calculated the ANI values for *R. leguminosarum*, *R. l. viciae, R. l. phaseoli*, and *R. etli* strains. The highest ANI value obtained for these strains was 92%. In a strict taxonomic classification, strains CCGM4, CCGM5, and CCGM6 should be renamed as *Rhizobium* sp.

Plasmids are important for bacteria to monitor and respond to environmental fluctuations [24]. Rhizobia possess several plasmids of high molecular weight, and these plasmids harbor approximately one-third of the total number of genes in the genome [25, 26]. In *R. etli*, genes for nodulation (*nod*) and nitrogen fixation (*nif*, *fix*) are arranged in clusters in the symbiotic plasmid, whereas other genes, such as the symbiotic cytochrome oxidase Cbb3 and transcriptional regulator *fixL* are reiterated in other plasmids [27]. Our functional analysis revealed that functions of transcription, synthesis of cofactors and metabolism of carbohydrates and amino acids were especially enriched in the seed strains (Fig. 4, Additional file 4). By contrast, nodule strains were enriched in hypothetical functions, possibly because these strains live in a more complex habitat.

Obligate symbionts undergo drastic genome reduction because some of their housekeeping functions are fulfilled by the host or because some defense mechanisms are no longer needed [28–30]. On the other hand, when an organism arrives in a new niche, it needs to adapt to the new environment and assimilate nutrients from new sources [31]. In this process, its genome may undergo expansions due to the duplication of existing genes, producing paralogs, or the acquisition of new ones through horizontal gene transfer [32]. Genomes of seed strains carried approximately 10% (i.e., 600) more genes than the typical nodule strains, suggesting gene gains (Table 1). We showed that paralogous genes were more abundant in seed strains, supporting the notion that the access to seeds represented a recent genomic expansion (Fig. 3d and e). In contrast, the functional reiteration was more abundant in the nodules strains CIAT652 and WSM2304. The gain of genes might represent the acquisition of new functions that improve the fitness of these strains in the new environment and are useful for their survival in the seeds. We propose that seed strains are in the stage of gene expansion. Despite the role of gene duplications in species diversification and the acquisition of new functions, this process can also lead to genomic instability [33].

About half of the genomes in bacterial databases contains genes of prophages [34], and the seed strains analyzed here showed several prophage sequences (Fig. 5). We showed that three of the seven strains, belonging to different rhizobial species, shared the same prophage sequence that was inserted exactly at the same genomic position in all three strains; this is rarely observed among bacterial genomes. Together with transposases, prophages participate in genomic rearrangements, loss of plasmids, recombination, and gain of genes [13]. We suggest that genomic sequences of prophages were inserted into those of the rhizobial strains after they infected the seeds, and participated in recombinant events and other rearrangements resulting in the loss of symbiosis genes. Given that the symbiotic capability of strains is not selected in the seeds, we wondered about the outcome of divergence process.

In *R. grahamii* strain CCGE502, 1 Mb of chromosomal DNA was homologous with a plasmid of CCGM3 (Fig. 2). Previous analysis of this genomic sequence showed that it is present in plasmids of closely related species, such as *R. mesoamericanum* STM3625, *R. tropici* CIAT899, and *R. etli* CFN42 [35]. The movement of large stretches of DNA between the chromosome and plasmids, including the symbiotic plasmid, has also been observed in other rhizobia, such as *Mesorhizobium huakuii*, *M. loti*, and *Bradyrhizobium* [36, 37]. This genomic rearrangement is possibly due to the action of transposases [38].

## Conclusions

The arrival of rhizobial strains into seeds of legume plants involved several genomic events such as expansion of gene families (paralogs), infection of phages, loss of plasmids, and rearrangement of replicons. The overall outcome of this process was the successful establishment of strains in this new niche. However, we detected ineffective sister strains due to the loss of genomic segments harboring genes necessary for nodulation and nitrogen fixation. Further research on similar microorganisms is needed to understand their dynamics with the plant and to explore their utility as seeds carrying their own inoculum.

## Methods

### Isolation of bacterial strains and culture medium

Rhizobial strains were isolated from root nodules of un-inoculated plants as described previously [8]. Strains were maintained in PY medium (0.5% peptone, 0.3% yeast extract, and 7 mM CaCl_2_).

### Greenhouse experiment

Seeds of common bean cultivar Negro Jamapa were surface sterilized with 20% sodium hypochloride for 5 min and rinsed extensively with sterile water. Seeds were then immersed in sterile water for 1 min and rinsed several times. For germination, seeds were arranged in a tray lined with moist paper and covered with aluminum foil. The tray was incubated in the dark for 3 days at 29 °C. Seedlings were transferred to sterile pots filled with vermiculite, and each plant was inoculated with 1 ml of freshly prepared bacterial suspension with an optical density at 540 nm (OD_540_) of 0.1. Plants were maintained in the greenhouse at a temperature ranging from 22 °C to 29 °C, 60% humidity, and natural photoperiod. Plants were irrigated alternately with sterile water and Fahraeus medium [39]. Plants un-inoculated with bacteria were used as controls; these plants were supplemented with nitrogen. Freshly harvested seeds were used for PCR.

### Polymerase chain reaction (PCR)

Seed extracts were prepared from fresh seeds that were mashed and resuspended in buffer. These extracts were then used for PCR. PCR was performed using T100 thermocycler and Phusion High-Fidelity DNA polymerase (ThermoScientific, Pittsburgh, PA, USA). PCR products were sequenced at the Sequencing Unit, Institute of Biotechnology-UNAM, Cuernavaca, México. Reaction conditions and primers used for PCRs are listed in Additional file 8.

### Plasmid visualization

The high molecular weight plasmids were visualized using the Eckhardt technique, as modified by Hynes and McGregor [40].

### Genome sequencing, assembly, and annotation

Genomic DNA was extracted from the rhizobial strains, following standard protocols [41], and used to prepare 3-kb DNA libraries. Samples were sequenced using Illumina MiSeq at Macrogen (Seoul, Korea) to obtain 100 bp mated-pair reads. Adapter sequences were removed from raw sequence reads using Trimmomatic v0.36 and from PhiX sequences using SMALT and ad-hoc perl scripts. Overlapping reads were joined using Flash v1.2.11. The genome statistics are summarized in Table 1 for each strain. Genome assembly was performed using Spades v3.10.0 [42] and Velvet v1.2.10 [43]. Gene annotation was performed using the NCBI Prokaryotic Genome Annotation Pipeline (PGAP). Functional classes were assigned using the cluster of orthologous genes COG classification (Galperin*,* et al., 2015).

### Comparative sequence analysis and detection of prophages

Pairwise comparisons were made using ProteinOrtho v5.15 [44] between strains that were previously shown to be closely related [8]. Blastn parameters were expectance less than 1 e (− 5), at least 60% identity and 70% of coverage. Rhizobial strains previously isolated from root nodules were included in the corresponding group, including *R. phaseoli* CIAT652, *R. leguminosarum* WSM2304, and *R. grahamii* CCGE502. The genome sequence of CCGM1 strain, which we previously characterized [9], was included in these comparisons, as its sequence is highly related to that of CCGM2. Prophages were identified using PHASTER [45]. The genome sequence of *R. leguminosarum* strain WSM1689 (accessions CP007045 to CP007050, corresponding to a chromosome and five plasmids) was also included in this analysis. Graph of prophages was generated using Circos [46].

### Accession numbers

The genomic sequences of each strain were deposited in the GenBank database under the provisional accession numbers shown in Table 1.

### Statistical analysis

Statistical differences were tested with the Fisher’s exact test.

## Additional files

Additional file 1: Detection of rhizobial strains in bean seeds via PCR amplifications. (PPTX 148 kb)

Additional file 2: List of genes present in strain CCGM5 and absent in CCGM4. (XLSX 189 kb)

Additional file 3: Average nucleotide identity (ANIm) and genome coverage of seed and nodule rhizobial strains. (PPTX 181 kb)

Additional file 4: Comparison of gene functions covered by seeds strains compared with nodule strains. (PPTX 206 kb)

Additional file 5: Shared and strain-specific genes in the analyzed rhizobial strains. (XLSX 455 kb)

Additional file 6: Genes with repeated function (by name). (XLSX 10 kb)

Additional file 7: Complementary data on the detection of prophages in the genomes of rhizobial strains. (XLSX 25 kb)

Additional file 8: Sequence of primers and PCR conditions. (DOCX 14 kb)

## Abbreviations

ANI: Average of nucleotide identity
*fix*: Nitrogen fixation genes
GTA: Gene transfer agent
*nif*: Nitrogen fixation genes
*nod*: Nodulation genes
OD: Optical density
PCR: Polymerase chain reaction
PY: Peptone-yeast medium
