# Supplementary material for: Analysis of genome sequence and symbiotic ability of rhizobial strains isolated from seeds of common bean (Phaseolus vulgaris)
Source: BMC Genomics. 2018 Aug 30;19:645. doi: 10.1186/s12864-018-5023-0 (PMC6117902; doi:10.1186/s12864-018-5023-0)
Supplement: Supplementary file 1 — Detection of rhizobial strains in bean seeds via PCR amplifications. (PPTX 148 kb) [file 12864_2018_5023_MOESM1_ESM.pptx]

## Slide 1
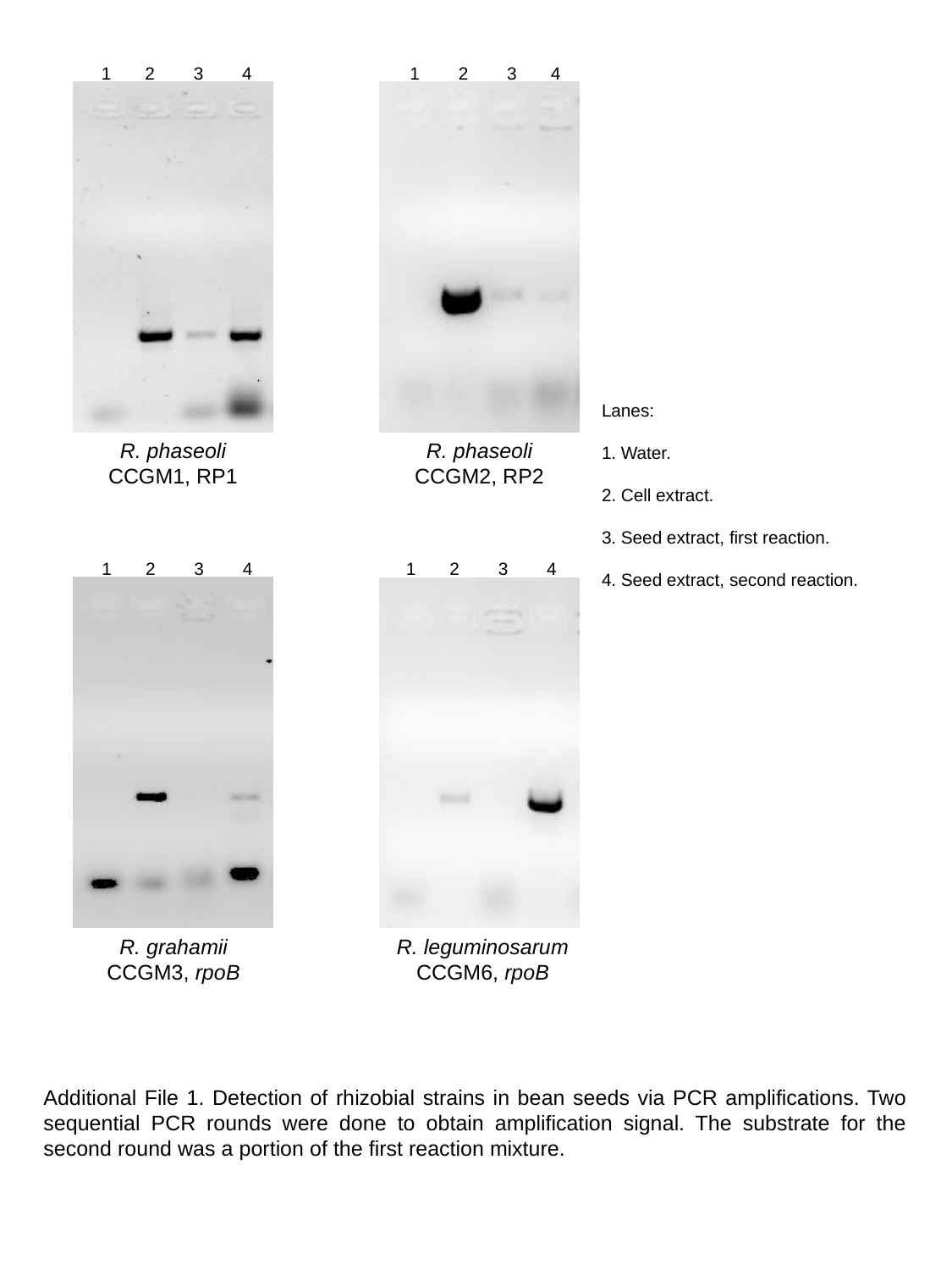

1 2 3 4
R. phaseoli
CCGM1, RP1
1 2 3 4
R. phaseoli
CCGM2, RP2
Lanes:
1. Water.
2. Cell extract.
3. Seed extract, first reaction.
4. Seed extract, second reaction.
1 2 3 4
R. grahamii
CCGM3, rpoB
1 2 3 4
R. leguminosarum
CCGM6, rpoB
Additional File 1. Detection of rhizobial strains in bean seeds via PCR amplifications. Two sequential PCR rounds were done to obtain amplification signal. The substrate for the second round was a portion of the first reaction mixture.
