# Supplementary material for: Analysis of genome sequence and symbiotic ability of rhizobial strains isolated from seeds of common bean (Phaseolus vulgaris)
Source: BMC Genomics. 2018 Aug 30;19:645. doi: 10.1186/s12864-018-5023-0 (PMC6117902; doi:10.1186/s12864-018-5023-0)
Supplement: Supplementary file 3 — Average nucleotide identity (ANIm) and genome coverage of seed and nodule rhizobial strains. (PPTX 181 kb) [file 12864_2018_5023_MOESM3_ESM.pptx]

## Slide 1
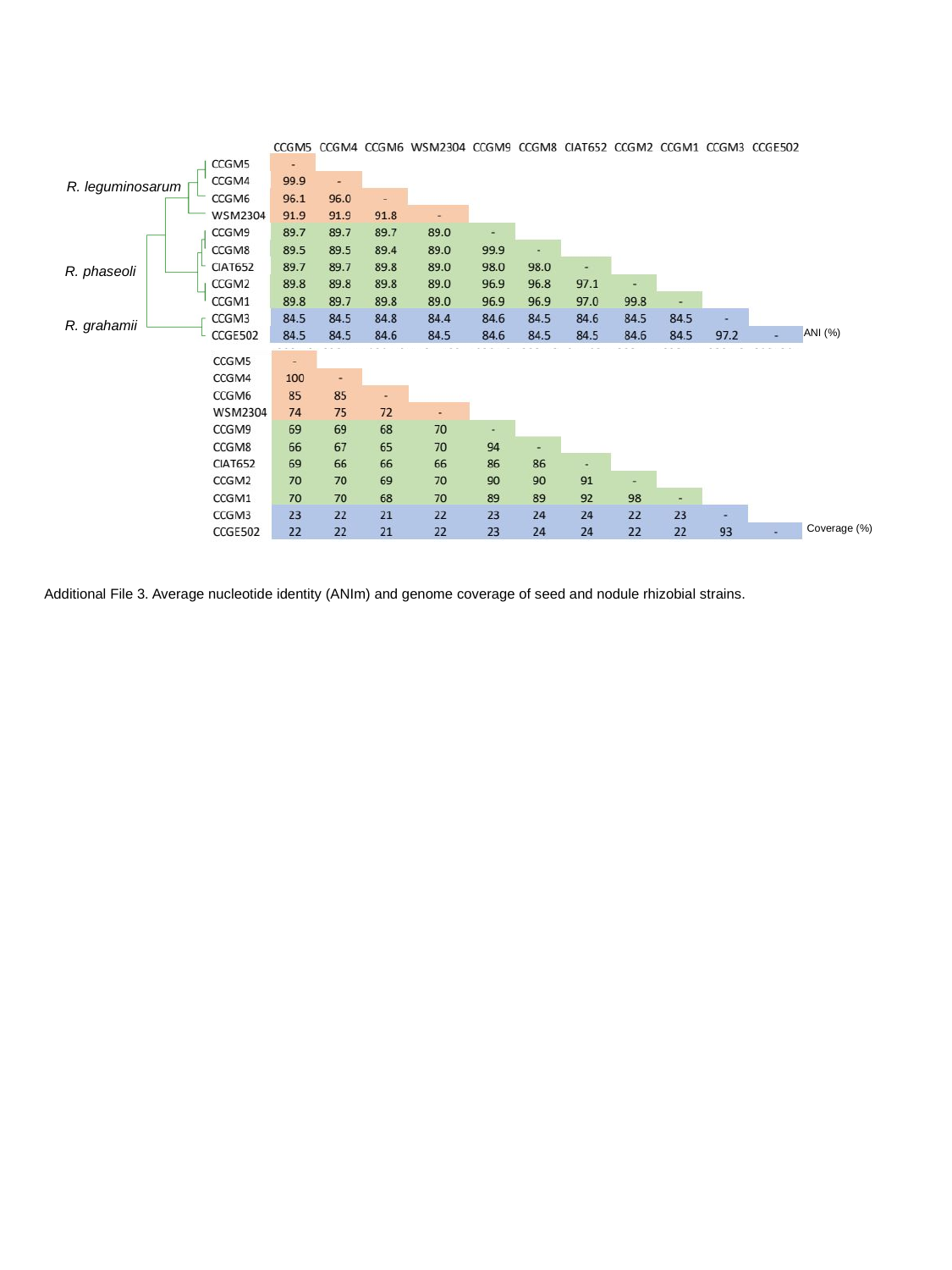

R. leguminosarum
R. phaseoli
R. grahamii
 ANI (%)
 Coverage (%)
Additional File 3. Average nucleotide identity (ANIm) and genome coverage of seed and nodule rhizobial strains.
