# Supplementary material for: Analysis of genome sequence and symbiotic ability of rhizobial strains isolated from seeds of common bean (Phaseolus vulgaris)
Source: BMC Genomics. 2018 Aug 30;19:645. doi: 10.1186/s12864-018-5023-0 (PMC6117902; doi:10.1186/s12864-018-5023-0)
Supplement: Supplementary file 4 — Comparison of gene functions covered by seeds strains compared with nodule strains. (PPTX 206 kb) [file 12864_2018_5023_MOESM4_ESM.pptx]

## Slide 1
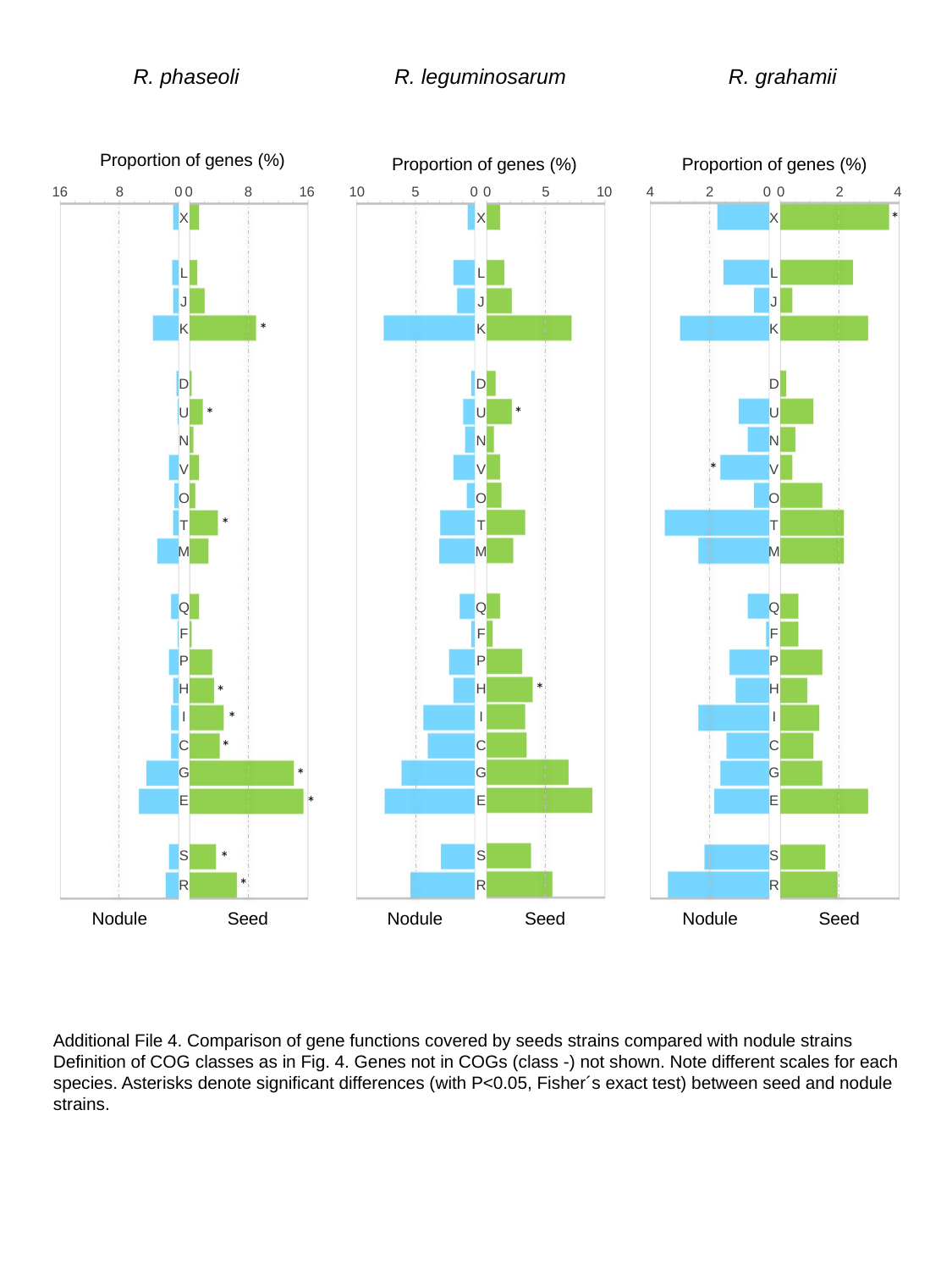

R. phaseoli
R. leguminosarum
R. grahamii
Proportion of genes (%)
Proportion of genes (%)
Proportion of genes (%)
10
5
0
0
5
10
X
L
J
K
D
U
N
V
O
T
M
Q
F
P
H
I
C
G
E
S
R
16
8
0
0
8
16
X
L
J
K
D
U
N
V
O
T
M
Q
F
P
H
I
C
G
E
S
R
4
2
0
0
2
4
X
L
J
K
D
U
N
V
O
T
M
Q
F
P
H
I
C
G
E
S
R
*
*
*
*
*
*
*
*
*
*
*
*
*
*
Nodule
Seed
Nodule
Seed
Nodule
Seed
Additional File 4. Comparison of gene functions covered by seeds strains compared with nodule strains Definition of COG classes as in Fig. 4. Genes not in COGs (class -) not shown. Note different scales for each species. Asterisks denote significant differences (with P<0.05, Fisher´s exact test) between seed and nodule strains.
