# Supplementary material for: Analysis of genome sequence and symbiotic ability of rhizobial strains isolated from seeds of common bean (Phaseolus vulgaris)
Source: BMC Genomics. 2018 Aug 30;19:645. doi: 10.1186/s12864-018-5023-0 (PMC6117902; doi:10.1186/s12864-018-5023-0)
Supplement: Supplementary file 8 — Sequence of primers and PCR conditions. (DOCX 14 kb) [file 12864_2018_5023_MOESM8_ESM.docx]

**Additional File 8.** Sequence of primers and PCR conditions.

| Strain specificity | Product  (size) | Primer name | Primer type | Primer sequence | PCR conditions | | |
| --- | --- | --- | --- | --- | --- | --- | --- |
|  |  |  |  |  | Initial step | Amplification  (35 cycles) | Final step |
| *R. phaseoli*  CCGM1 | Glycosyl transferase  RLPCCGM1_c4056  (661 bp) | RP1_fw | Forward | GGTCCGTGAACAGGCAAAG | 15s at 95ºC | 2s at 98º, 2s at 59º,  20s at 72º | 20s at 72º |
|  |  | RP1_rv | Reverse | GCAGCCGCATTTGTTCGTTAA |  |  |  |
| *R. phaseoli*  CCGM2 | Hypothetical protein  B5K03_14135  (530 bp) | RP2_fw | Forward | CTTGCTCCGGCACTCTCTAAGG | 15s at 95ºC | 2s at 98º, 2s at 56º,  20s at 72º | 20s at 72º |
|  |  | RP2_rv | Reverse | GTTCTCGGGATCGCAGATGC |  |  |  |
| R. *grahamii*  CCGM3  *R. leguminosarum*  CCGM6 | RNA polymerase  *rpoB*  (786 bp) | *rpoB*_RSM_fw | Forward | ACCCGCGATATTCCGAAYGTHTC | 15s at 95ºC | 2s at 98º, 2s at 58º,  2s at 55º, 25s at 72º | 25s at 72º |
|  |  | *rpoB*_RSM_rv | Reverse | GTTCAGAACGACGTCGACRTG |  |  |  |
